# Genetic Variation of Blast (*Pyricularia oryzae* Cavara) Resistance in the Longistaminata Chromosome Segment Introgression Lines (LCSILs) and Potential for Breeding Use in Kenya

**DOI:** 10.3390/plants12040863

**Published:** 2023-02-14

**Authors:** Rena Tomita, Vincent Pamugas Reyes, Yoshimichi Fukuta, Emily Waringa Gichuhi, Mayumi Kikuta, Daniel Makori Menge, Kazuyuki Doi, Daigo Makihara

**Affiliations:** 1 Graduate School of Bioagricultural Sciences, Nagoya University, Nagoya 464-8601, Japan; 2 Japan International Research Center for Agricultural Sciences, Tsukuba 305-8686, Japan; 3 Kenya Agricultural and Livestock Research Organization, Kerugoya P.O. Box 298-10300, Kenya; 4 Graduate School of Integrated Sciences for Life, Hiroshima University, Higashi-Hiroshima 739-8528, Japan; 5 International Center for Research and Education in Agriculture, Nagoya University, Nagoya 464-8601, Japan

**Keywords:** basmati, blast (*Pyricularia oryzae* Cavara), CSSL, disease resistance, genetic variation, Kenya, *Oryza longistaminata*, rice (*Oryza sativa* L.)

## Abstract

In Kenya’s rice-growing areas, Basmati varieties have been produced in monoculture since the late 1980s. This has resulted in the breakdown of the resistance (R) gene-mediated response of the local Basmati varieties to blast disease caused by *Pyricularia oryzae*. To improve blast resistance in Kenyan Basmati varieties, continuous identification of R genes and suitable breeding materials for Basmati are necessary. Longistaminata chromosome segment introgression lines (LCSILs) with the Kernel Basmati genetic background, developed using a rice line called potential low-input adaptable-1 (pLIA-1) derived from a cross between Taichung 65 (T65) (a rice variety in the Japonica Group) and *O. longistaminata*, are expected to contain useful blast R genes derived from *O. longistaminata* or T65. In this study, we investigated the genetic variation of blast R genes in LCSILs and their parents by using a new international differential system for designating blast races based on the gene-for-gene theory and molecular characterization using single nucleotide polymorphism (SNP) markers. LCSILs and their parents were classified into three groups—A, B1, and B2—based on reaction patterns to the standard differential blast isolates (SDBIs). Group A, including pLIA-1, showed the highest resistance in all groups, followed by groups B1 and B2. Kernel Basmati in group B1 was considered to possess *Pik-p* or *Pi7*(t), *Pi19*(t), and other unknown R genes. In addition to these R genes, LCSIL 6, 12, 27, 28, and 40, in group A, were determined to possess one of *Pish*, *Piz-t*, or both genes that confer resistance to the Kenyan blast races. These lines can be used for efficiently pyramiding blast R genes in the local Basmati varieties.

## 1. Introduction

In sub-Saharan Africa, the demand for rice has been increasing in recent decades [1]. In Kenya, rice consumption has been rising with population growth and changes in eating habits [2,3]. Kenya imported 250 million USD of rice in 2020 [1]. Thus, domestic rice production must be expanded along with the increased demand. However, rice productivity in Kenya is suppressed by various biotic and abiotic stresses [4]. Among them, rice blast disease is the major constraint to rice production [5,6,7]. In the Mwea irrigation scheme, producing more than 80% of rice in Kenya, the main variety grown since the late 1980s has been Basmati [3,4,5]. In the Kenyan market, Basmati rice is preferred, and its price is approximately twice as high as that of non-aromatic rice [8]. However, several studies have reported outbreaks of blast disease in the Basmati variety in Kenya [5,9]. The breeding of Basmati varieties for blast resistance has not progressed much, mainly because of the hybrid sterility between Basmati varieties and japonica or indica rice [10]. Therefore, it is important to improve blast resistance in the Basmati variety.

Rice blast disease caused by *Pyricularia oryzae* Cavara is one of the most devastating diseases in rice. Host resistance is considered the major and economically viable approach for managing this disease. Host resistance to blast commonly follows the gene-for-gene theory, where a single dominant resistance (R) gene effectively controls the infection of strains carrying the corresponding avirulence (AVR) gene [11,12]. Hundreds of rice blast R genes have been identified, and 38 have been cloned [13]. Several recent studies have utilized some of these genes to develop rice lines with improved resistance to blast [14,15,16,17]. However, blast pathogens can overcome the R gene-mediated resistance due to the yearslong intensive selection pressure of a monocropped single-resistance cultivar and the emergence of new pathotypes [18]. To address this issue, continuous identification of R genes is necessary. 

It is necessary to determine the pathogenicity of blast isolates in the target area and resistance in the rice variety to develop blast-resistant varieties. Hayashi & Fukuta [19] developed a new international differential system for designating races of the rice blast pathogen based on the gene-for-gene theory. This system consists of international differential varieties (DVs) and standard differential blast isolates (SDBIs). DVs are 25 monogenic lines targeting 23 R genes, including *Pia*, *Pish*, *Pib*, *Pit*, *Pii*, *Pi3*, *Pi5*(t), *Pik-s*, *Pik*, *Pik-p*, *Pi7*(t), *Pik-m*, *Pi1*, *Pik-h*, *Piz*, *Piz-5*, *Piz-t*, *Pi9*(t), *Pi19*(t), *Pi20*(t), *Pita*, *Pita-2*, and *Pi12*(t) with the genetic background of a susceptible Chinese Japonica Group rice variety, Lijiangxintuanheigu (LTH), which has no major R gene. Fukuta [20] successfully selected several SDBIs from collections in Asia and African counties. Using international DVs and the designation system of blast races, we can determine the differentiation of blast races in the field and evaluate the blast R genes in rice varieties.

Wild rice species are considered an untapped source of valuable traits that could be exploited for improving cultivated rice. Several studies have reported their importance in providing genes for resistance to insects and viruses and tolerance to drought stress [21,22,23]. Several breeding populations, such as chromosomal segment substitution lines (CSSLs), have been developed using different rice species in the cultivated rice *O. sativa* L. background [24,25,26,27,28,29]. CSSLs are considered pre-breeding genetic stocks that can be utilized for faster and easier development of near-isogenic lines (NILs). In addition, CSSLs are also suitable for quantitative trait loci (QTL) analysis because each line has minimal introgression and their effects are not masked by other segments [30,31]. Longistaminata chromosome segment introgression lines (LCSILs) are CSSL populations with the Kernel Basmati genetic background developed using a rice line called potential low-input adaptable-1 (pLIA-1), derived from a cross between the Japonica Group rice variety Taichung 65 (T65) and *O. longistaminata* [32,33]. LCSILs are expected to contain useful blast R genes derived from *O. longistaminata* or T65. 

Recent advances in sequencing technology have led to the development of different genotyping techniques. Recently, genotyping-by-sequencing (GBS) has been successfully implemented across various practices in plant genetics, such as the development of mapping populations, QTL mapping, and marker-assisted introgression of beneficial QTL [34,35,36,37]. Unlike the gel-based genotyping approach, sequencing-based high-throughput methods such as GBS are more efficient and cost-effective. Gichuhi et al. [33,38] have characterized the genomic composition of LCSILs and pLIA-1 using SSR markers. However, only 18 SSR markers were used for genotyping the pLIA-1, and only 85 were used for genotyping the LCSIL population. High-resolution mapping using thousands of markers should be able to identify *O. longistaminata* segments that were not detected in the previous study by Gichuhi et al. [33]. 

In this study, we aimed to identify the genomic regions associated with blast resistance in LCSILs. To achieve this, we evaluated the blast resistance of LCSILs based on the international differential system. Using GBS, we obtained high-resolution genotypes of LCSILs and evaluated R genes that were associated with blast resistance. Elite lines, together with the genetic information of QTL(s) for enhancing blast resistance in Kenyan Basmati varieties, were obtained based on these results. 

## 2. Results

### 2.1. Molecular Characteristics of LCSILs and pLIA-1

**Figure 1:**
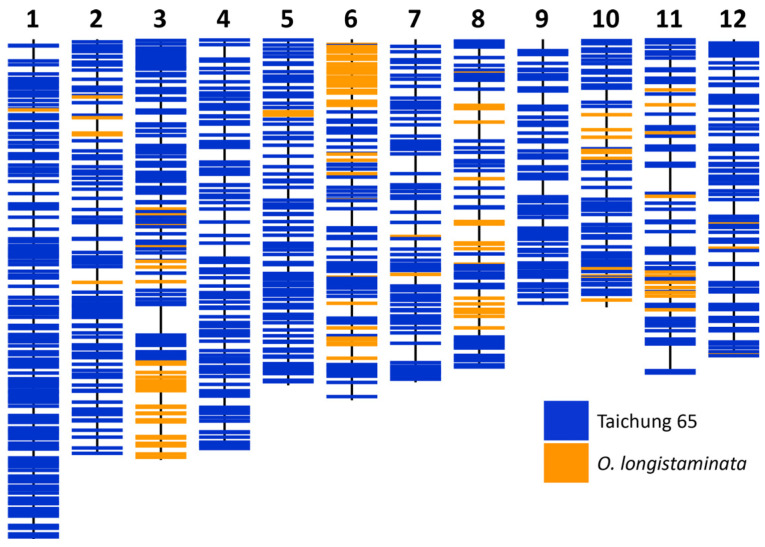
A total of 1285 SNP markers in the donor line, pLIA-1. Blue and orange bars indicate the genomes of the Japonica Group variety T65 and the wild rice *O. longistaminata*, respectively.

The molecular characterization of pLIA-1 and LCSILs was carried out using the information obtained from the GBS analysis. Using 1285 single nucleotide polymorphisms (SNPs), the parental line, pLIA-1, was observed to have 88.8% of the T65 genome, 11.0% of the *O. longistaminata* genome, and 0.2% missing information. The *O. longistaminata* chromosomal segments were identified on chromosomes (chr.) 1, 2, 3, 5, 6, 7, 8, 10, 11, and 12 (Figure 1). The chromosomal segments from *O. longistaminata* in the pLIA-1 ranged from 0.0–24.5%, with chr. 6 having the highest percentage introgression. *Oryza longistaminata* chromosomal segments were uniquely identified at the 6.6 Mb region of chr. 2, the 15.1 Mb region of chr. 3, the 6.4 Mb region of chr. 5, and the 21.6 Mb region of chr. 11 of pLIA-1. 

**Figure 2:**
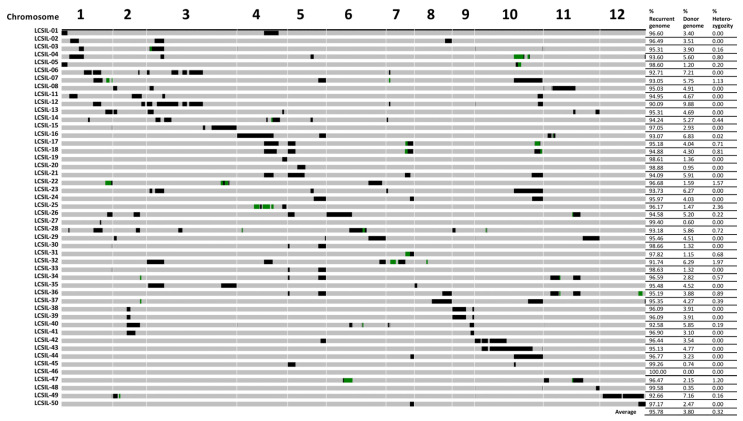
Graphical genotype and percentage of genotypic composition of LCSILs based on the total of 1705 SNP markers. The diagram on the left shows the introgression of pLIA-1 alleles (black) in the genetic background of Kernel Basmati (gray). Green bars represent heterozygous regions. The right panel shows the percentage of the number of markers of recurrent (Kernel Basmati), donor (pLIA-1), and heterozygous markers.

As for LCSILs, 48 lines were characterized using 1705 high-quality SNPs (Figure 2). The graphical genotype of the LCSILs is presented in Figure 2. Using these informative markers, we identified chromosomal regions that have pLIA-1 segments. The genotyped LCSILs carried an average of 95.7% Kernel Basmati genome, 3.9% pLIA-1 genome, and 0.3% heterozygous segments. A total of 159 donor segments were detected in the LCSILs, and 128 were evenly distributed in 12 chromosomes. The LCSIL population used in this study followed the theoretical percentage for a recurrent parent genome (93.8%) at the BC_3_ generation. 

There were 13 lines of LCSILs in which regions of R genes carried by DVs were substituted with pLIA-1. The substitution segments in LCSIL 6, 12, 27, and 28 were located at the *Pish* locus; LCSIL 1 and 5 at the *Pit* locus; LCSIL 16, 34, and 36 at the *Pia* locus; LCSIL 29 and 48 at the *Pik* locus; and LCSIL 26, 28, and 40 at the *Piz* locus. In addition, the substitution segment in LCSIL 8 was located at the *pi21*(t) locus [39]. 

### 2.2. Classification of LCSILs Based on the Blast Resistance Phenotyping

**Figure 3:**
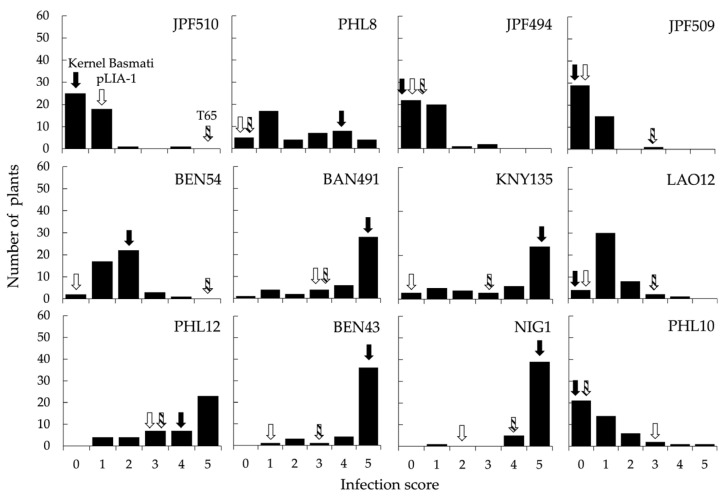
Frequency distribution of infection scores to SDBIs in LCSILs. Solid, open, and hatched arrows indicate infection scores of Kernel Basmati, pLIA-1, and T65, respectively.

LCSILs and their parents showed different reaction patterns to SDBIs (Table S1). Some of the LCSILs scored quite differently to Kernel Basmati, which is their genetic background (Figure 3). The LCSILs’ donor line, pLIA-1, showed high or moderate resistance to the SDBIs used for testing. In detail, pLIA-1 showed increased resistance to two blast races, similarly to what was observed in Kernel Basmati, and higher resistance than Kernel Basmati to eight SDBIs. 

**Figure 4:**
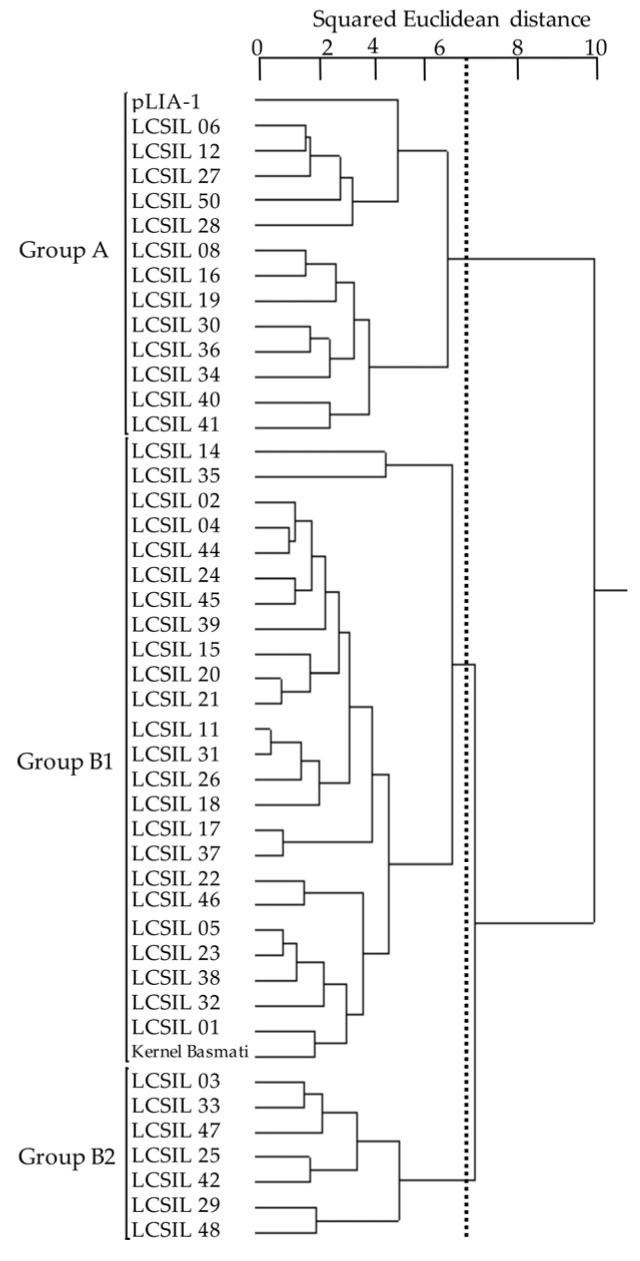
Classification of LCSILs based on the resistance spectrum to SDBIs. A total of 46 lines, including parents, Kernel Basmati, and pLIA-1, were classified into three cluster groups, A, B1, and B2.

Cluster analysis for the infection scores to the 12 SDBIs showed that LCSILs, Kernel Basmati, and pLIA-1 were classified into two clusters, group A and group B (Figure 4). Group B was further classified into group B1 and group B2. Group A included 13 LCSILs and pLIA-1, group B1 included 24 LCSILs and Kernel Basmati, and group B2 included seven LCSILs.

There were differences among the three clusters in infection scores for all blast isolates except JPF494 and JPF509 (Table 1). The mean score was lowest in group A and highest in group B2. Group A showed higher resistance than group B1 to seven SDBIs and higher resistance than group B2 to six SDBIs. Furthermore, group B1 showed higher resistance to the three SDBIs than group B2.

### 2.3. Evaluation of Blast Resistance Genes

Based on a comparative analysis of the response patterns of the infection scores of the DVs and the tested rice lines to SDBIs, all LCSILs are expected to possess several R genes (Table S1). Table 2 shows the results of the evaluation of blast R genes for each cluster group and the parents of LCSILs. Kernel Basmati was presumed to possess either *Pik-p* or *Pi7*(t) and unknown gene(s). Although pLIA-1 was assumed to contain unidentified blast R genes, this differential system could not infer the specific genes because it showed resistance to all 12 SDBIs. T65 was presumed to possess *Pish* and unknown gene(s). The presumed genotypes of LCSILs showed different trends for each group classified by cluster analysis. Most of the LCSILs classified in group A were presumed to possess one of the *Pish*, *Pib*, *Pia*, and *Pii* double alleles, one of the *Pik* double alleles, *Piz-t*, *Pi20*(t), and unknown gene(s). The LCSILs classified as group B1 were presumed to possess one of *Pik-m*, *Pik-p* or *Pi7*(t), and unknown gene(s), while group B2 was presumed to contain one of *Pik-m*, *Pik-h*, *Pik-p*, or *Pi7*(t) and unknown gene(s). However, LCSIL 29 and 48, classified as group B2, were the only LCSILs not presumed to possess *Pik* double alleles among all LCSILs.

### 2.4. QTL Mapping of Blast Resistance Loci in LCSILs

Using the SNP and phenotypic data obtained with the 12 SDBIs, QTL analysis for blast resistance was carried out and resulted in the identification of 14 blast resistance QTL (*qBR*) (LOD > 3.0) in the rice genome (Figure S1). The *qBR*s were identified on chr. 1, 2, 3, 4, 7, 8, 11, and 12, wherein phenotypic variance explained (PVE) by each QTL ranged from 27.8% to 69.9%. Among the 14 *qBR*s, four (*qBR1.1*, *qBAL8*, *qBR4*, and *qBR11.2*) had a PVE greater than 50.0% and an LOD score > 6.0. The major QTL *qBR4* was identified at the 20.5 Mb region of chr. 4 with an LOD score of 11.7 and 69.9% PVE and was observed to be associated with the NIG1 isolate. The *qBR11.2*, associated with the PHL10 isolate, was identified at the 28.0 Mb region of chr. 11. This QTL had an LOD score of 7.9 and 55.8% PVE. The major QTL *qBR1.2*, associated with the BEN43 blast isolate, was identified at the 32.5 Mb region of chr. 1 and had an LOD score of 7.2 and 52.5% PVE. Lastly, the major QTL *qBR8* was placed at the 211 Kb region of chr. 8 and was observed to be associated with JPF494. This QTL had an LOD score of 7.2 and 50.1% PVE. Several other *qBR*s were identified in our study with PVE (%) < 50.0%. For example, several *qBR*s that were associated with the NIG1 blast isolate and mapped to chr. 1 (*qBR1.1*), 3 (*qBR3*), and 7 (*qBR7*) were identified. Each accounted for 31.3% of PVE. On chr. 11, the *qBR11.1*, which was observed to be associated with the KNY135 blast isolate, was mapped to the 7.3 Mb region. This QTL had an LOD score of 3.7 and accounted for 31.8% PVE. Similarly, *qBR11.2*, associated with the LAO12 blast isolate, was mapped at 21.4 Mb. This QTL accounted for 37.2% PVE and had an LOD score of 4.5. The *qBR12* identified at the 27 Mb region was observed to be associated with BEN43 and accounted for 31.1% PVE. 

The summary of the identified *qBR*s is presented in Table 3. We observed co-localization of resistance in regions of chr. 1 and 11. For example, *qBR*s associated with PHL12, BEN49, and BEN43 were co-localized in the 32.5 Mb region of chr. 1. Similarly, *qBR*s associated with LAO12 and PHL10 were co-localized in the 28.0 Mb region of chr. 11.

## 3. Discussion

### 3.1. Detection of Blast Resistance Genotypes of LCSILs and Their Parents

Blast R genes in LCSILs could be assigned using the international differential system. We validated and further narrowed them down by comparing them with the results of genetic analyses. The reaction patterns of most LCSILs to SDBIs were similar to those of Kernel Basmati (Figure 4). The differences between the lines were attributed to the introduction of chromosome segments from the donor parent, pLIA-1. 

In the study by Gichuhi et al. [33], an average of one introgression was observed in the LCSILs, whereas in this study, an average of three donor segments per line was observed, likely because a larger number of markers were utilized. The presence of substitutions with chromosomal segments of pLIA-1 in the blast resistance locus region determined using the international differential system matched the locus regions of the *Pish*, *Pia*, *Pik* double alleles, and the *Piz* double alleles in the ten LCSILs (Table 4). This result indicates that LCSIL 6, 12, 27, and 28 are likely to possess *Pish*; LCSIL 16, 34, and 36 have *Pia*; all lines except LCSIL 29 and 48 have *Pik* double alleles; and LCSIL 28 and 40 have *Piz* double alleles.

LCSIL 6, 12, 27, and 28, presumed to possess *Pish*, were classified as group A and showed strong or moderate resistance to all blast races except NIG1. The *Pish* locus region of the donor parent, pLIA-1, was most likely derived from T65 (Figure 1 [38]). T65 has been reported to possess *Pish* [40,41] and was found to likely contain *Pish* in this study (Table 2).

LCSIL 16, 34, and 36, presumed to possess *Pia*, were classified as group A with strong resistance. The *Pia* locus region on chr. 11 of pLIA-1 was derived from either T65 or *O. longistaminata* (Figure 1 [38]). Our results suggested that T65 was not likely to possess *Pia*, which was consistent with the results of previous studies [40,41]. Therefore, *Pia* was most likely derived from *O. longistaminata*. 

In LCSIL 29 and 48, the region containing the *Pik* double alleles was substituted with pLIA-1, and these lines were presumed to be the only LCSILs without either of the *Pik* double alleles. Thus, Kernel Basmati, the genetic background of LCSILs, was considered to possess one of the *Pik* double alleles, whereas pLIA-1 did not. Fukuta et al. [7] found that Basmati 217 and Basmati 370 contained one of the *Pik* double alleles. Furthermore, Kim et al. [42] found that many Basmati varieties, including Basmati 370, possessed *Pik*, *Pik-m*, and *Pik-p* using SNP markers. Therefore, Kernel Basmati was considered to have *Pik-p* or *Pi7*(t).

LCSIL 28 and 40, presumed to have *Piz-t*, were classified as group A with strong blast resistance. The region containing the *Piz-t* locus on chr. 6 of pLIA-1 was derived from T65 or *O. longistaminata* (Figure 1 [38]). T65 was presumed not to possess *Piz* double alleles (Table S1 [40,41]). Furthermore, chr. 6, the locus of *Piz-t*, had the highest percentage of *O. longistaminata* substitutions (Figure 1). Therefore, *Piz-t*, which was presumed to be present in LCSIL 28 and 40 in this study, was likely to be derived from *O. longistaminata.* In addition to LCSIL 28 and 40, the region containing the *Piz* locus of LCSIL 26 was also substituted with pLIA-1. However, unlike the other two lines, LCSIL 26 was classified as group B1, which was not in the resistant group. This may be because the expression of the introgressed resistant gene was affected by the length of introgression segments and other resistant genes in the genetic background [43,44]. 

Analysis of response patterns in the rice blast inoculation test either showed that all LCSILs and Kernel Basmati possess *Pi19*(t), one of the *Pita* double alleles, or that the presence of *Pi19*(t) was masked by other R genes (Table S1). Furthermore, in this study, the region of the *Pita* double alleles was not substituted in all LCSILs. Therefore, it was reasonable to assume that all LCSILs and Kernel Basmati have *Pi19*(t). This was supported by the fact that Basmati 217 and Basmati 370 were thought to include *Pi20*(t), one of the *Pita* double alleles [7].

Considering the results of the genetic analysis and the evaluation of R genes based on the response patterns in the rice blast inoculation test, the parental variety Kernel Basmati was considered to possess *Pi19*(t) and unknown genes in addition to *Pik-p* or *Pi7*(t), while pLIA-1 contained at least *Pish*, *Pia,* and *Piz-t* but no *Pik* double alleles. Fukuta et al. [7] reported that Basmati 217 and Basmati 370 were classified as the most resistant group of the varieties grown in Kenya to the Japanese, Philippine, and Kenyan blast races, and they were presumed to possess *Pi20*(t), one of the *Pik* double alleles or *Pi3*, and unknown gene(s). Therefore, the parental variety Kernel Basmati was considered to have a similar level of resistance to Basmati 217 and Basmati 370 among the varieties grown in Kenya. However, in areas where Basmati varieties are widely grown, they are generally known to be susceptible to blast [45,46,47]. In Kenya, Basmati varieties have been reported to be severely damaged by blast [5,6,7]. In these areas, blast races that can infect Basmati varieties are dominant because Basmati varieties have been grown for many years [7,48]. 

### 3.2. Blast Resistance Genes That May Be Effective in Kenya 

There have been several reports on effective blast R genes in Kenya. Nyongesa et al. [8] reported that rice lines carrying *Piz-t* and *Pita-2* showed resistance in three rice-growing areas of Kenya, Embu, Kisumu, and Tana River, while *Pik-s*, *Pik-h*, *Piz-5*, *Piz*, *Pit*, *Pish*, *Pi1*, *Pi5*(t), *Pik-m*, *Pita-2*, *Pib*, and *Pik* were resistant in Embu and Tana River. Fukuta et al. [48] reported that lines carrying *Pi9* were resistant to blast races in five rice-growing regions: Embu, Kisumu, Tanah River, Kirinyaga, and Mombasa. Similarly, lines with *Pish*, *Pib*, *Pit*, *Pik-s*, *Pik-m*, *Pi1*, *Pik-h*, *Pik*, *Pik-p*, *Pi7*(t), *Piz*, *Piz-5*, *Piz-t*, *Pita-2*, *Pita*, *Pi12*(t), and *Pi19*(t) were resistant in some regions. However, *Pii*, *Pi3*, and *Pi5*(t) were considered at high risk of resistance breakdown in five regions, and *Pit*, *Pia*, *Pib*, *Pik-s*, *Pik-m*, *Pi1*, *Pik-h*, *Pik*, *Pik-p*, *Pi7*(t), *Pi12*(t), *Pita*, *Pi19*(t), and *Pi20*(t) were at risk of resistance breakdown in four regions.

In this study, LCSILs and their parents were presumed to contain *Pik-p* or *Pi7*(t), *Pish*, *Pia*, *Piz-t*, *Pi19*(t), and unknown R genes. Among these, *Piz-t* has been reported to show resistance to many blast races when introduced into susceptible lines [49,50,51]. *Piz-t* showed resistance in more than three Kenya regions and is considered a stable R gene. *Pish*, which showed resistance in some regions, was also considered a useful gene with high stability. On the other hand, *Pik-p*, *Pi7*(t), and *Pi19*(t) are at increased risk of resistance breakdown and may become susceptible to blast within a few years. LCSIL 28, which showed good resistance in this study, was presumed to possess *Pish*, *Pik-p* or *Pi7*(t), *Piz-t*, and *Pi19*(t) (Table 4), as well as R genes other than the 23 genes in the DVs. In addition, LCSIL 26 and 40, presumed to harbor *Piz-t* and LCSIL 6, 12, and 27, presumed to possess *Pish*, would be useful breeding material for blast-resistant Basmati varieties for Kenya. Furthermore, LCSIL 8, 19, 36, 41, and 50, classified as Group A, showed good resistance to many SDBIs. However, their reaction patterns differed from those of the DVs, suggesting that their blast resistance was conferred by genes other than the 23 R genes introduced in the DVs. Among them, LCSIL 8, in which pLIA-1 was introduced into the *pi21*(t) locus region on chr. 4 [39], may possess *pi21*(t) from T65 (Figure 1 [38]).

This study detected 14 rice blast resistance QTLs, including *qBR1.2*, *qBR1.3*, and *qBR1.4* for resistance to BEN43, PHL12, and BAN491, and *qBR11.3* and *qBR11.4* for resistance to PHL10 and LAO12, which were identified in the regions containing *Pish* and *Pik* double alleles, respectively. Furthermore, *qBR11.1* for resistance to KNY135 was identified near the *Pia* locus. In addition, *qBR4* for resistance to NIG1 was identified near *pi21*(t) [39], although it is not included in the R genes of the DVs. The QTLs *qBR3* and *qBR7* convey resistance to NIG1, and *qBR12* conveys resistance to BEN43. LCSILs 14 and 50 substituted with pLIA-1 in these regions showed strong resistance to the respective blast races. Thus, *qBR3*, *qBR7*, and *qBR12* likely contain new R genes that have not yet been reported. Since pLIA-1, the donor parent of the LCSILs, contains chromosome segments of *O. longistaminata* (Figure 1 [38]), some of the novel blast R genes may be derived from *O. longistaminata*. Further research is needed to identify these novel blast-resistant genes.

### 3.3. Potential for Breeding Use of LCSILs

Blast resistance in Basmati has not been reported as much as in japonica varieties. Moreover, the breeding of blast-resistant Basmati varieties is not as advanced as with japonica varieties. One of the major challenges in breeding Basmati varieties is hybrid sterility. Hybrid sterility is known to occur in crosses between japonica and indica varieties [52,53], and Basmati varieties have been reported to be hybrid sterile with japonica and indica varieties [10]. 

To date, some blast-resistant lines have been produced by introducing *Pi9*(t) into Ranbir Basmati with a genetic background of Basmati 370 [47]; *Pi2* and *Pi54* into Pusa Basmati 1121 and Pusa Basmati 6 [54]; and *Pi2* (=*Piz-5*), *Pi9*(t), *Pi1*, *Pi54*, *Pita*, *Pib*, and *Pi5*(t) into Pusa Basmati 1 [46]. However, these lines are not available in Kenya. Few useful materials are available for breeding for blast resistance in Basmati. Using LCSIL 6, 12, 27, 28, and 40, which have Kernel Basmati genetic backgrounds, as breeding material for crosses, it will be possible to develop blast-resistant Basmati for Kenya carrying *Piz-t* and *Pish* without causing hybrid sterility. LCSILs other than these selected lines can also introduce other R genes such as *Pia*, *Pik* double alleles, etc. In addition, LCSILs were found to possess several unknown R gene(s), some of which were likely derived from the African wild rice *O. longistaminata*. Further research is needed to investigate the potential breeding use of these unknown R gene(s).

## 4. Materials and Methods

### 4.1. Plant Materials

**Figure 5:**
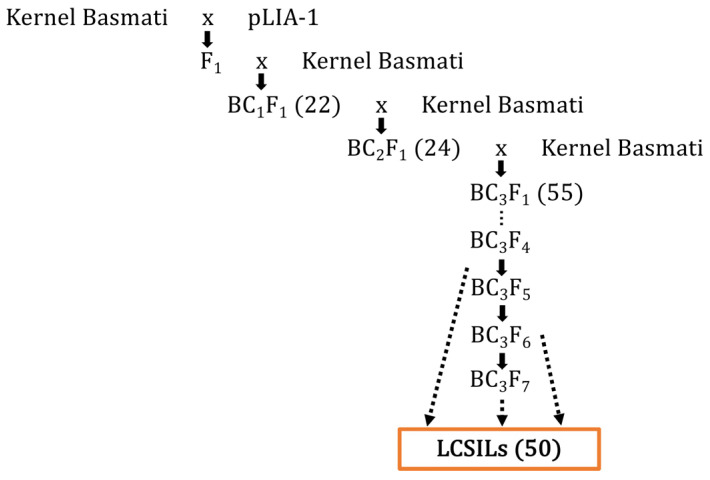
LCSILs breeding scheme. A total of 50 lines at BC_3_F_5_, BC_3_F_6_, and BC_3_F_7_ generations were selected for LCSILs, as shown by dashed arrows.

The breeding population used in the study is comprised of LCSILs, which have the genetic background of Kernel Basmati, and part of the chromosome has been replaced by a pLIA-1 chromosome [33]. pLIA-1 is a rice line derived from an interspecific cross between the wild rice species, *O. longistaminata*, and a rice cultivar, T65 [32]. It was initially described to have a large biomass even when grown under non-fertilized conditions [33,38,55]. On the other hand, Kernel Basmati is an aromatic rice variety with excellent grain quality. In summary, F_1_ hybrids were backcrossed to the recurrent parent, Kernel Basmati, to obtain BC_1_F_1_ plants. A total of 22 plants were selected, and successive backcrossing was conducted to obtain BC_2_F_1_ plants. In the BC_3_F_1_ generation, a total of 55 plants were selected and selfed. A total of 50 lines were selected at the BC_3_F_5_, BC_3_F_6_, and BC_3_F_7_ generations (Figure 5). 

### 4.2. Genotyping

Plant materials, including 48 lines of LCSILs, parental lines of LCSILs, Kernel Basmati, and pLIA-1 were grown in the paddy field at the Higashiyama campus of Nagoya University, Nagoya, Japan, in 2020. They were sown in early June and transplanted in late June. The leaf materials from three plants of each line were harvested in late November and dried at 56 °C for 24 h.

The GBS library for the LCSILs was prepared following the protocol of Poland et al. [56] and Furuta et al. [57]. In summary, genomic DNA (200 ng) of plant material was extracted using the cetyltrimethylammonium bromide (CTAB) method [58]. The DNA quality and quantity were analyzed using a 1% agarose gel and Quantus TM Fluorometer (Promega, Madison, WI, USA). The DNA concentration of each sample was then adjusted to 20 ng/uL and double-digested using a combination of restriction enzymes *Kpn*I-*Msp*I. The double-digested DNA samples were ligated with unique barcode adapters and pooled into a single tube. The library sequencing was carried out using Illumina Miseq (Illumina, Inc., San Diego, CA, USA). 

The informatics processing of the GBS reads was conducted using the TASSEL-GBS 5.0 pipeline [59]. In summary, the obtained 64 bp long sequence tags were aligned to the IRGSP V1.0 *O. sativa* Nipponbare reference genome sequence [60] using the Burrows-Wheeler Aligner (BWA) [61]. The SNPs were called by filtering the minimum allele frequency (mnMAF) to 0.02 and the minimum locus coverage (mnLCov) to 0.8. The obtained reads were further filtered based on polymorphism between parental alleles using an awk script written by the authors, and the remaining low-quality markers were manually removed. 

### 4.3. Phenotyping for Blast Resistance

Blast resistance of 45 lines of LCSILs, Kernel Basmati, pLIA-1 and T65 were evaluated with 28 DVs, including 25 DVs that included monogenic lines and NILs, by inoculation tests targeting 23 R genes [19]. In this study, monogenic lines IRBL5-M, IRBL1-CL, IRBLkh-K3, IRBLk-Ka, IRBLta2-Pi, and IRBLta-K1 were replaced with six near-isogenic lines (NIL): IRBL5-M[LT] for *Pi5*(t); IRBL1-CL[LT] for *Pi1*; IRBLkh-K3[LT] for *Pik-h*; IRBLk-Ka[LT] for *Pik*; IRBLta2-Pi[LT] for *Pita-2*; and IRBLta-K1[LT] for *Pita,* each with the genetic background of the susceptible Chinese Japonica Group rice variety, Lijiangxintuanheigu (LTH) [62]. Moreover, three NILs were used: IRBL5-M[US] for *Pi5*(t); IRBL12-M[US] for *Pi12*(t); and IRBLta-K1[US] for *Pita,* each with the genetic background of the susceptible Indica Group rice variety US-2 [63]. LTH and US-2 were evaluated as the susceptible control variety. 

In total, 12 SDBIs were used, including three from Japan (JPF494, JPF509, JPF510) (Mu-95, Ina93-3, GFOS8-1-1) [64], three from the Philippines (PHL10, PHL8, PHL12) (Ca41, M39-1-3-8-1, M64-1-3-9-1) [65], one from Bangladesh (BAN491) (BD1092) [66], one from Laos (LAO12) (H08-259-1) [67], three from west Africa (BEN43, BEN54, NIG1) (Bn93, OUED10.4.5, NI1) (unpublished), and one from Kenya (KNY135) (15ke69) [48].

Stock isolates were cultured on an oatmeal agar medium and incubated at around 25 °C for 12 to 13 days. The culture plate was scraped using a toothbrush and exposed to fluorescent light for 4 to 5 days to induce heavy sporulation. Using a paintbrush, conidia were dislodged from the plate into 10 to 20 mL of sterilized distilled water containing 0.01% of tween 20. Spore suspensions were filtered, and spore concentration was adjusted to 1 × 10^5^ conidia per mL using a hemacytometer [68]. Plants were grown in a greenhouse for 2 weeks (approximately 4- to 5-leaf stage) and inoculated by spraying with a spore suspension. After inoculation, the seedlings were incubated at 25 °C at a relative humidity of 70 to 80% for 20 h and then transferred to a greenhouse at a relative humidity of approximately 60% and a temperature of around 25 °C. 

The infection score of each isolate was evaluated based on the 6-rating system 7 days after inoculation [69]. The experiment was performed twice. For evaluating the R genes, a score of 0 to 2 was categorized as resistant (R), 2.5 to 3 as moderate resistance (M), and 3.5 to 5 as susceptible (S). By comparing the DVs’ reaction patterns to the SDBIs, we evaluated the resistance genes for LCSILs and parental lines and varieties.

### 4.4. QTL Analysis for Blast Resistance

The QTL analysis was performed by CSL function and single-marker analysis (SMA) implemented in the QTL IciMapping ver. 4.1 [70]. The threshold LOD value was determined by a permutation test involving 1000 runs at a significance level of *p*  =  0.05, and the QTL in a particular genomic region with LOD values larger than this threshold for each trait were used. 

### 4.5. Statistical Analysis

Cluster analysis using Ward’s hierarchical method was performed for the classification of introgression lines to SDBIs. The infection scores of all lines were analyzed using the Shapiro–Wilk normality test. Since these replicated data did not show a normal distribution, the nonparametric Steel–Dwass test was performed following a Kruskal–Wallis test to evaluate the differences between the groups classified by cluster analysis. All statistical analyses were performed using the averages of two inoculation experiments by JMP ver. 16 (SAS Institute, Inc., Cary, NC, USA).

## 5. Conclusions

To control blast in Kenya without excessive reliance on pesticides, it will be necessary to develop new varieties carrying multiple blast R genes, or to grow multiple varieties with different R genes, including multilines (a mixture of near-isogenic lines with varying sources of resistance). However, few reports of such measures being applied in developing countries are available. Furthermore, relevant studies using Basmati varieties are extremely limited. In this study, blast resistance phenotypes and genotypes of LCSILs were evaluated by the international differential system and genetic analysis, and lines showing high resistance to the Kenyan blast races were selected. The selected lines are LCSIL 6, 12, 27, 28, and 40, which can be used for efficiently pyramiding blast R genes in the local Basmati varieties. They will also be useful in developing multilines with different blast R genes. Thus, the findings of this study can provide a significant contribution to overcoming rice blast disease in Kenya.

## Figures and Tables

**Table 1:** Infection score of each group classified by reaction patterns to SDBIs.

Cluster Group (No. of Lines)	Infection Score												
	Standard Differential Blast Isolates (SDBIs)												
	JPF510	PHL8	JPF494	JPF509	BEN54	BAN491	KNY135	LAO12	PHL12	BEN43	NIG1	PHL10	Mean
Group A (14)	0.2 b	0.7 b	0.1 a	0.1 a	0.9 b	2.5 b	1.0 b	0.7 b	2.6 b	3.4 b	4.4 b	0.8 ab	1.4 c
Group B1 (25)	0.2 b	2.1 a	0.4 a	0.2 a	1.6 a	4.6 a	4.6 a	0.7 b	4.1 a	4.9 a	4.8 a	0.3 b	2.4 b
Group B2 (7)	0.8 a	3.3 a	0.4 a	0.4 a	1.3 ab	4.4 ab	4.2 a	1.9 a	4.9 a	5.0 ab	5.0 ab	1.9 a	2.7 a
Total (46)	0.4	2.0	0.3	0.2	1.3	3.8	3.3	1.1	3.9	4.4	4.7	1.0	2.2
	**	**	n.s.	n.s.	**	***	***	*	**	*	**	**	***

*, ** and ***: significant at *p* < 0.05, 0.01 and 0.001 by Kruskal–Wallis test; n.s.: not significant at *p* < 0.05 by Kruskal–Wallis test. Different letters indicate valuables are significantly different between cluster groups at *p* < 0.05 by Steel–Dwass test.

**Table 2:** Evaluation of putative blast resistance genes for each resistance group shown in Figure 4.

Cluster Group /Varieties/Lines	Expected Resistance Genes
A	*Pish*, *Pib*, *Pia*, one of *Pii* alleles, one of *Pik* alleles, *Piz-t*, *Pi20*(t), Unknown(s)
B1	one of *Pik-m*, *Pik-p* or *Pi7*(t), Unknown(s)
B2	one of *Pik-m*, *Pik-h*, *Pik-p* or *Pi7*(t) (no *Pik* alleles in 2 lines), Unknown(s)
Kernel Basmati	one of *Pik-p* or *Pi7*(t), Unknown(s)
pLIA-1	Indecipherable, Unknown(s)
Taichung 65	*Pish*, Unknown(s)

**Table 3:** QTL associated with blast resistance in LCSILs.

QTL	Isolate	Chr ^a^	Marker Name	Position (Mb)	LOD	PVE (%) ^b^	AE ^c^
*qBR1.1*	NIG1	1	S01_23977952	24.0	3.6	31.3	−0.9
*qBR1.2*	BEN43	1	S01_32539855	32.5	7.2	52.5	−1.4
*qBR1.3*	PHL12	1	S01_32539855	32.5	5.2	41.9	−1.6
*qBR1.4*	BAN491	1	S01_32539855	32.5	3.1	27.9	−1.3
*qBR2*	LAO12	2	S02_1819274	1.8	3.1	27.8	0.8
*qBR3*	NIG1	3	S03_15189839	15.2	3.6	31.3	−0.9
*qBR4*	NIG1	4	S04_20521698	20.5	11.7	69.9	−1.9
*qBR7*	NIG1	7	S07_810885	0.8	3.6	31.3	−0.9
*qBR8*	JPF494	8	S08_211700	0.2	6.7	50.1	1.1
*qBR11.1*	LAO12	11	S11_7355421	7.4	3.7	31.8	−1.7
*qBR11.2*	PHL10	11	S11_21408258	21.4	4.5	37.2	1.0
*qBR11.3*	LAO12	11	S11_28067973	28.1	7.9	55.8	1.8
*qBR11.4*	KNY135	11	S11_28067973	28.1	3.1	27.8	0.8
*qBR12*	BEN43	12	S12_27046865	27.0	3.6	31.1	−2.1

^a^ Chromosome. ^b^ Percentage of variance explained by QTL. ^c^ Additive effects.

**Table 4:** Evaluation of blast resistance gene(s) in LCSIL lines with corresponding genotype and their reaction patterns to SDBIs.

Varieties /Lines	Locus	Cluster Group	Reaction Patterns											
			Standard Differential Blast Isolates (SDBIs)											
			JPF510	PHL8	JPF494	JPF509	BEN54	BAN491	KNY135	LAO12	PHL12	BEN43	NIG1	PHL10
LCSIL 6	*Pish*	A	R	R	R	R	R	R	R	R	R	R	S	R
LCSIL 12	*Pish*	A	R	R	R	R	R	M	R	R	R	M	S	R
LCSIL 16	*Pia*	A	R	R	R	R	R	S	R	R	S	S	S	R
LCSIL 27	*Pish*	A	R	R	R	R	R	R	R	R	R	R	S	R
LCSIL 28	*Pish, Piz*	A	R	R	R	R	R	R	R	R	R	R	S	M
LCSIL 34	*Pia*	A	R	R	R	R	R	S	R	R	S	S	S	R
LCSIL 36	*Pia*	A	R	R	R	R	R	M	R	R	R	S	S	R
LCSIL 40	*Piz*	A	R	R	R	R	R	R	R	R	R	S	S	R
LCSIL 29	*Pik*	B2	R	R	R	R	R	M	S	M	S	S	S	S
LCSIL 48	*Pik*	B2	R	S	R	R	R	S	S	R	S	S	S	S
Kernel Basmati	-	B1	R	S	R	R	R	S	S	R	S	S	S	R
pLIA-1	-	A	R	R	R	R	R	M	R	R	M	R	R	M

R, M, and S indicate resistant, moderate-resistant, and susceptible.

## Data Availability

Data generated in this study is available upon request to the corresponding author.

## Supplementary Materials

The following are available online at: https://www.mdpi.com/article/10.3390/plants12040863/s1. Table S1: Response patterns of LCSILs, Kernel Basmati, pLIA-1, T65, and international differential varieties (DVs) to 12 standard differential blast isolates (SDBIs). Figure S1: SNP markers used for QTL mapping and position of 14 blast resistance QTLs (*qBR*) (LOD > 3.0).

## References

[1] FAOSTAT. https://faostat.fao.org/.

[2] Atera E.A., Onyango J.C., Azuma T., Asanuma S., Itoh K. (2011). Field evaluation of selected NERICA rice cultivars in western Kenya. Afr. J. Agric. Res..

[3] Mati B.M., Wanjogu R., Odongo B., Home P.G. (2011). Introduction of the System of Rice Intensification in Kenya: Experiences from Mwea Irrigation Scheme. Paddy Water Environ..

[4] Onyango A.O. (2014). Exploring options for improving rice production to reduce hunger and poverty in Kenya. World Environ..

[5] Kihoro J., Bosco N.J., Murage H., Ateka E., Makihara D. (2013). Investigating the impact of rice blast disease on the livelihood of the local farmers in greater Mwea region of Kenya. SpringerPlus.

[6] Kariaga M.G., Wakhungu J., Were H.K. (2016). Identification of rice blast (*Pyricularia oryzae* Cav.) races from Kenyan rice growing regions using culture and classical characterization. J. Res. Agric. Anim. Sci..

[7] Fukuta Y., Suzuki T., Yanagihara S., Obara M., Tomita A., Ohsawa R., Machungo C.W., Hayashi N., Makihara D. (2019). Genetic variation of blast (*Pyricularia oryzae* Cavara) resistance in rice (*Oryza sativa* L.) accessions widely used in Kenya. Breed. Sci..

[8] Atera E.A., Onyancha F.N., Majiwa E.B.O. (2018). Production and marketing of rice in Kenya: Challenges and opportunities. J. Dev. Agric. Econ..

[9] Nyongesa B.O., Bigirimana J., Were B.A., Murori R. (2016). Virulence spectrum of populations of *Pyricularia oryzae* in irrigated rice ecosystems in Kenya. Eur. J. Plant Pathol..

[10] Zhang Y., Wang J., Pu Q., Yang Y., Lv Y., Zhou J., Li J., Deng X., Wang M., Tao D. (2022). Understanding the nature of hybrid sterility and divergence of Asian cultivated rice. Front. Plant Sci..

[11] Flor H.H. (1971). Current status of the gene-for-gene concept. Annu. Rev. Phytopathol..

[12] Silué D. (1992). Evidence of a gene-for-gene relationship in the *Oryza sativa-Magnaporthe grisea* Pathosystem. Phytopathology.

[13] Hu Z.J., Huang Y.Y., Lin X.Y., Feng H., Zhou S.X., Xie Y., Liu X.X., Liu C., Zhao R.M., Zhao W.S. (2022). Loss and natural variations of Blast Fungal avirulence genes breakdown rice resistance genes in the Sichuan Basin of China. Front. Plant Sci..

[14] Angeles-Shim R.B., Reyes V.P., del Valle M.M., Lapis R.S., Shim J., Sunohara H., Jena K.K., Ashikari M., Doi K. (2020). Marker-assisted introgression of quantitative resistance gene *pi21* confers broad spectrum resistance to rice blast. Rice Sci..

[15] Khan G.H., Shikari A.B., Vaishnavi R., Najeeb S., Padder B.A., Bhat Z.A., Parray G.A., Bhat M.A., Kumar R., Singh N.K. (2018). Marker-assisted introgression of three dominant blast resistance genes into an aromatic rice cultivar Mushk Budji. Sci. Rep..

[16] Koide Y., Kobayashi N., Xu D., Fukuta Y. (2009). Resistance genes and selection DNA markers for Blast Disease in rice (*Oryza sativa* L.). Jpn. Agric. Res. Q. JARQ.

[17] Yang D., Tang J., Yang D., Chen Y., Ali J., Mou T. (2019). Improving rice blast resistance of Feng39S through molecular marker-assisted backcrossing. Rice.

[18] Valent B., Khang C.H. (2010). Recent advances in rice blast effector research. Curr. Opin. Plant Biol..

[19] Hayashi N., Fukuta Y. (2009). Proposal for a New International System of Differentiating Races of Blast (*Pyricularia oryzae* Cavara) by Using LTH Monogenic Lines in Rice (*Oryza sativa* L.). JIRCAS Work. Rep..

[20] Fukuta Y., Fukuta Y., Hasebe A., Kato M., Yang R.Y. (2021). JIRCAS Blast Research Network for Stable Rice Production. Applicable Solution Against Rice Blast in Asia.

[21] Brar D., Khush G., Kang M.S. (2002). Transferring genes from wild species into rice. Quantitative Genetics, Genomics and Plant Breeding.

[22] Brar D.S., Khush G.S., Sasaki T., Moore G. (1997). Alien introgression in rice. Oryza: From Molecule to Plant.

[23] Jena K., Khush G., Nanda J.S. (2000). Exploitation of species in rice improvement-opportunities, achievements and future challenges. Rice Breeding and Genetic: Research Priorities and Challenges.

[24] Doi K., Iwata N., Yoshimura A. (1997). The construction of chromosome substitution lines of African rice (*Oryza glaberrima* Steud.) in the background of japonica rice (*O. sativa* L.). Rice Genet. Newsl..

[25] Furuta T., Uehara K., Angeles-Shim R.B., Shim J., Ashikari M., Takashi T. (2014). Development and evaluation of chromosome segment substitution lines (CSSLs) carrying chromosome segments derived from *Oryza rufipogon* in the genetic background of *Oryza sativa* L.. Breed. Sci..

[26] Furuta T., Uehara K., Angeles-Shim R.B., Shim J., Nagai K., Ashikari M., Takashi T. (2016). Development of chromosome segment substitution lines harboring *Oryza nivara* genomic segments in Koshihikari and evaluation of yield-related traits. Breed. Sci..

[27] Ramos J.M., Furuta T., Uehara K., Chihiro N., Angeles-Shim R.B., Shim J., Brar D.S., Ashikari M., Jena K.K. (2016). Development of chromosome segment substitution lines (CSSLs) of *Oryza longistaminata* A. Chev. & Röhr in the background of the elite japonica rice cultivar, Taichung 65 and their evaluation for yield traits. Euphytica.

[28] Shim R.A., Angeles E.R., Ashikari M., Takashi T. (2010). Development and evaluation of *Oryza glaberrima* Steud. chromosome segment substitution lines (CSSLs) in the background of *O. sativa* L. cv. Koshihikari. Breed. Sci..

[29] Yoshimura A., Nagayama H., Sobrizal, Karakazu T., Sanchez P.L., Doi K., Yamagata Y., Yasui H. (2010). Introgression lines of rice (*Oryza sativa* L.) carrying a donor genome from the wild species, *O. glumaepatula* Steud. and *O. meridionalis* Ng. Breed. Sci..

[30] Ali M.L., Sanchez P.L., Yu S., Lorieux M., Eizenga G.C. (2010). Chromosome segment substitution lines: A powerful tool for the introgression of valuable genes from *Oryza* Wild species into cultivated rice (*O. sativa*). Rice.

[31] Balakrishnan D., Surapaneni M., Mesapogu S., Neelamraju S. (2019). Development and use of chromosome segment substitution lines as a genetic resource for crop improvement. Theor. Appl. Genet..

[32] Gichuhi E., Himi E., Takahashi H., Maekawa M. (2012). Oryza longistaminata’s chromosome segments are responsible for agronomically important traits for environmentally smart rice. Proceedings of the 7th JKUAT Scientific, Technological and Industrialization Conference.

[33] Gichuhi E., Himi E., Ahmed N., Takahashi H., Maekawa M. (2016). Preliminary QTL detection for improving basmati rice in a F_2_ population derived from the cross between kernel basmati and pLIA-1 carrying *Oryza longistaminata* chromosome. SABRAO J. Breed. Genet..

[34] Arbelaez J.D., Moreno L.T., Singh N., Tung C.W., Maron L.G., Ospina Y., Martinez C.P., Grenier C., Lorieux M., McCouch S. (2015). Development and GBS-genotyping of introgression lines (ILs) using two wild species of rice, *O. meridionalis* and *O. rufipogon*, in a common recurrent parent, *O. sativa* cv. Curinga. Mol. Breed..

[35] Kitony J.K., Sunohara H., Tasaki M., Mori J.I., Shimazu A., Reyes V.P., Yasui H., Yamagata Y., Yoshimura A., Yamasaki M. (2021). Development of an Aus-derived nested association mapping (Aus-NAM) population in rice. Plants.

[36] Phung H.D., Sugiura D., Sunohara H., Makihara D., Kondo M., Nishiuchi S., Doi K. (2019). QTL analysis for carbon assimilate translocation-related traits during maturity in rice (*Oryza sativa* L.). Breed. Sci..

[37] Reyes V.P., Angeles-Shim R.B., Mendioro M.S., Manuel M.C.C., Lapis R.S., Shim J., Sunohara H., Nishiuchi S., Kikuta M., Makihara D. (2021). Marker-assisted introgression and stacking of major QTLs controlling grain number (*Gn1a*) and number of primary branching (*WFP*) to NERICA cultivars. Plants.

[38] Gichuhi E., Himi E., Takahashi H., Maekawa M. (2016). Characterization and QTL Analysis of *Oryza longistaminata* Introgression Line, pLIA-1, derived from a Cross between *Oryza longistaminata* and Oryza sativa (Taichung 65) under Non-fertilized Conditions. J Rice Res..

[39] Fukuoka S., Okuno K. (2001). QTL analysis and mapping of *pi21*, a recessive gene for field resistance to rice blast in Japanese upland rice. Theor. Appl. Genet..

[40] Kawasaki-Tanaka A., Fukuta Y. (2014). Genetic variation in resistance to blast disease (*Pyricularia oryzae* Cavara) in Japanese rice (*Oryza sativa* L.), as determined using a differential system. Breed. Sci..

[41] Kitazawa N., Shomura A., Mizubayashi T., Ando T., Nagata K., Hayashi N., Takahashi A., Yamanouchi U., Fukuoka S., Yamanouchi U. (2019). Rapid DNA-genotyping system targeting ten loci for resistance to blast disease in rice. Breed. Sci..

[42] Kim J.S., Ahn S.N., Kim C.K., Shim C.K. (2010). Screening of rice blast resistance genes from aromatic rice germplasms with SNP markers. Plant Pathol. J..

[43] Cesari S., Thilliez G., Ribot C., Chalvon V., Michel C., Jauneau A., Rivas S., Alaux L., Kanzaki H., Okuyama Y. (2013). The rice resistance protein pair RGA4/RGA5 recognizes the *Magnaporthe oryzae* effectors AVR-Pia and AVR1-CO39 by direct binding. Plant Cell..

[44] Tsunematsu H., Yanoria M.J.T., Ebron L.A., Hayashi N., Ando I., Kato H., Imbe T., Khush G.S., Khush G.S. (2000). Development of monogenic lines of rice for blast resistance. Breed. Sci..

[45] Gouda P.K., Saikumar S., Varma C.M.K., Nagesh K., Thippeswamy S., Shenoy V., Ramesha M.S., Shashidhar H.E. (2013). Marker-assisted breeding of *Pi-1* and *Piz-5* genes imparting resistance to rice blast in PRR 78, restorer line of Pusa RH-10 Basmati rice hybrid. Plant Breed..

[46] Khanna A., Sharma V., Ellur R.K., Shikari A.B., Gopala Krishnan S., Singh U.D., Prakash G., Sharma T.R., Rathour R., Variar M. (2015). Development and evaluation of near-isogenic lines for major blast resistance gene(s) in Basmati rice. Theor. Appl. Genet..

[47] Samal P., Pote T.D., Krishnan S.G., Singh A.K., Salgotra R.K., Rathour R. (2019). Integrating marker-assisted selection and doubled haploidy for rapid introgression of semi-dwarfing and blast resistance genes into a Basmati rice variety ‘Ranbir Basmati’. Euphytica.

[48] Fukuta Y., Telebanco-Yanoria M.J., Hayashi N., Yanagihara S., Machungo C.W., Makihara D. (2019). Pathogenicities of rice blast (*Pyricularia oryzae* Cavara) isolates from Kenya. Plant Dis..

[49] Akator S.K., Adjata D.K., Drissa S., Awande S., Zadji L., Sangare G., Sere Y., Gumedzoe Y.M.D. (2013). Pathological studies of *Pyricularia oryzae* at m’be in Coted’Ivoire and Ouedeme in Benin. Asian J. Plant Pathol..

[50] Ghaley B.B., Christiansen J.L., Andersen S.B. (2012). Genetic diversity in blast resistance of Bhutan rice landraces. Euphytica.

[51] Odjo T., Ahohuendo B.C., Onasanya A., Akator K., Séré Y. (2011). Analysis of *Magnaporthe oryzae* structure in Benin. Afr. J. Agric. Res..

[52] Kato S., Kosaka H., Hara S. (1928). On the affinity of rice varieties as shown by the fertility of hybrid plants. Bull. Sci..

[53] Liu K.D., Zhou Z.Q., Xu C.G., Zhang Q., Saghai Maroof M.A. (1996). An analysis of hybrid sterility in rice using a diallel cross of 21 parents involving indica, japonica and wide compatibility varieties. Euphytica.

[54] Ellur R.K., Khanna A., Yadav A., Pathania S., Rajashekara H., Singh V.K., Gopala Krishnan S., Bhowmick P.K., Nagarajan M., Vinod K.K. (2016). Improvement of Basmati rice varieties for resistance to blast and bacterial blight diseases using marker assisted backcross breeding. Plant Sci..

[55] Gichuhi E., Himi E., Takahashi H., Zhu S., Doi K., Tsugane K., Maekawa M. (2016). Identification of QTLs for yield-related traits in RILs derived from the cross between pLIA-1 carrying *Oryza longistaminata* chromosome segments and Norin 18 in rice. Breed. Sci..

[56] Poland J.A., Brown P.J., Sorrells M.E., Jannink J.L. (2012). Development of high-density genetic maps for barley and wheat using a novel two-enzyme genotyping-by-sequencing approach. PLoS ONE.

[57] Furuta T., Ashikari M., Jena K.K., Doi K., Reuscher S. (2017). Adapting genotyping-by-sequencing for rice F2 populations. G3 Genes Genomes Genet..

[58] Doyle J.J., Doyle J.L. (1987). A rapid DNA isolation procedure for small quantities of fresh leaf tissue. Phytochem. Bull..

[59] Bradbury P.J., Zhang Z., Kroon D.E., Casstevens T.M., Ramdoss Y., Buckler E.S. (2007). TASSEL: Software for association mapping of complex traits in diverse samples. Bioinformatics.

[60] Kawahara Y., de la Bastide M., Hamilton J.P., Kanamori H., McCombie W.R., Ouyang S., Schwartz D.C., Tanaka T., Wu J., Zhou S. (2013). Improvement of the *Oryza sativa* Nipponbare reference genome using next generation sequence and optical map data. Rice.

[61] Li H., Durbin R. (2009). Fast and accurate short read alignment with Burrows-Wheeler transform. Bioinformatics.

[62] Telebanco-Yanoria M.J., Koide Y., Fukuta Y., Imbe T., Kato H., Tsunematsu H., Kobayashi N. (2010). Development of near-isogenic lines of Japonica-type rice variety Lijiangxintuanheigu as differentials for blast resistance. Breed. Sci..

[63] Fukuta Y., Koide Y., Kobayashi N., Kato H., Saito H., Telebanco-Yanoria M.J., Ebron L.A., Mercado-Escueta D., Tsunematsu H., Ando I. (2022). Lines for blast resistance genes with genetic background of Indica Group rice as international differential variety set. Plant Breed..

[64] Hayashi N. (2005). Rice Blast Fungus, MAFF Microorganism Genetic Resources Manual No. 18.

[65] Telebanco-Yanoria M.J., Imbe T., Kato H., Tsunematsu H., Ebron L.A., Vera Cruz C.M.V., Kobayashi N., Fukuta Y., Fukuta Y. (2008). A set of standard differential blast isolates (*Magnaporthe grisea* (Hebert) Barr.) from the Philippines for rice (*Oryza sativa* L.) resistance. Jpn. Agric. Res. Q. JARQ.

[66] Khan M.A.I., Ali M.A., Monsur M.A., Kawasaki-Tanaka A., Hayashi N., Yanagihara S., Obara M., Mia M.A.T., Latif M.A., Fukuta Y. (2016). Diversity and distribution of rice blast (*Pyricularia oryzae* Cavara) races in Bangladesh. Plant Dis..

[67] Xangsayasane P., Boualaphanh C., Bounphanousay C., Bounphanousay V., Manivong P., Voradeth S., Inthapanya P., Sato T., Obara M., Hayashi N. (2020). Genetic variation of rice blast (*Pyricularia oryzae*) isolates in Laos. Plant Health Prog..

[68] Hayashi N., Ando I., Imbe T. (1998). Identification of a new resistance gene to a Chinese blast fungus isolate in the Japanese rice cultivar Aichi Asahi. Phytopathology.

[69] Hayashi N., Kobayashi N., Cruz C.M.V., Fukuta Y. (2009). Protocols for the Sampling of Diseased Specimens and Evaluation of Blast Disease in Rice. JIRCAS Work. Rep..

[70] Meng L., Li H., Zhang L., Wang J. (2015). QTL IciMapping: Integrated software for genetic linkage map construction and quantitative trait locus mapping in biparental populations. Crop J..

