# Supplementary figures and images for: Genetic Variation of Blast (Pyricularia oryzae Cavara) Resistance in the Longistaminata Chromosome Segment Introgression Lines (LCSILs) and Potential for Breeding Use in Kenya
Source: Plants (Basel). 2023 Feb 14;12(4):863. doi: 10.3390/plants12040863 (PMC9966461; doi:10.3390/plants12040863)

Chr. 1

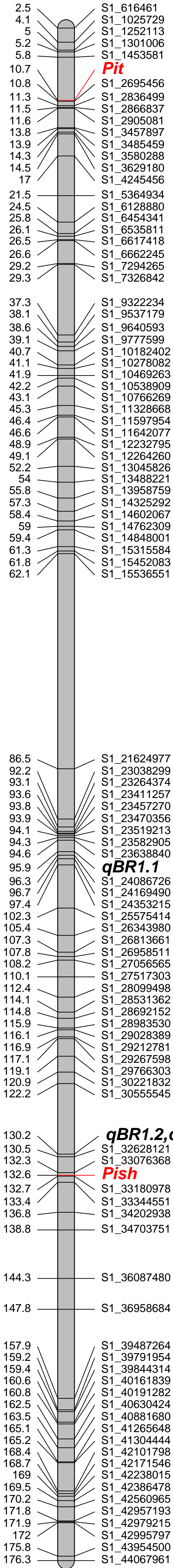

Chr. 2

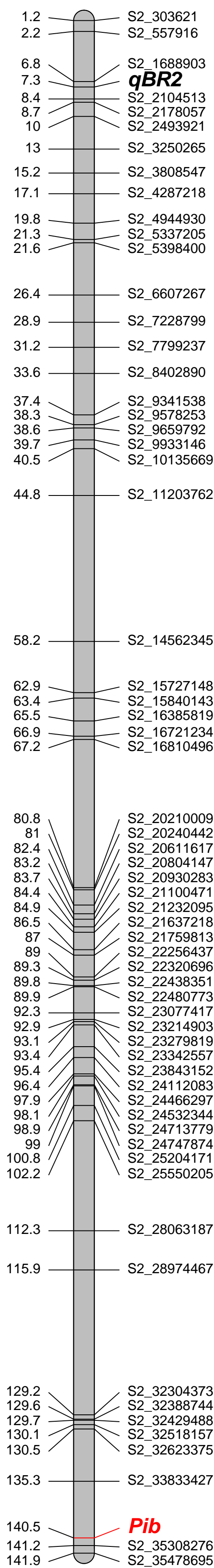

Chr. 3

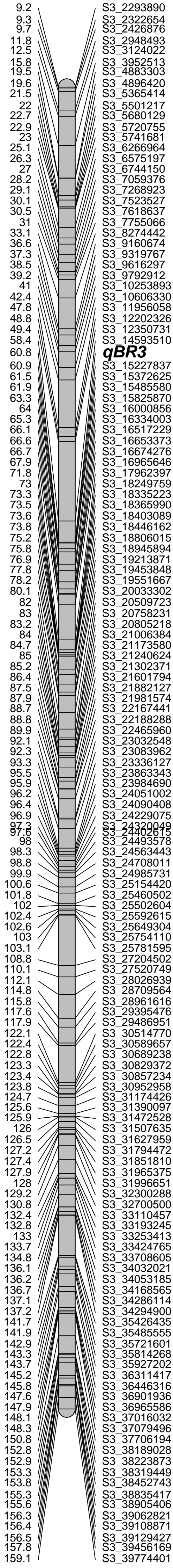

Chr. 4

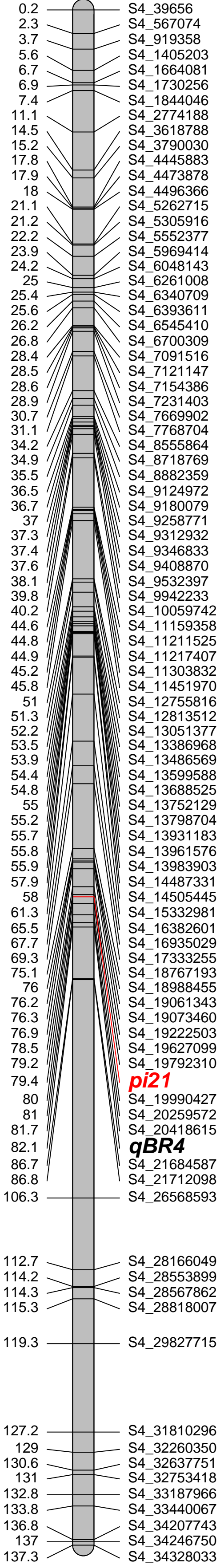

Chr. 7

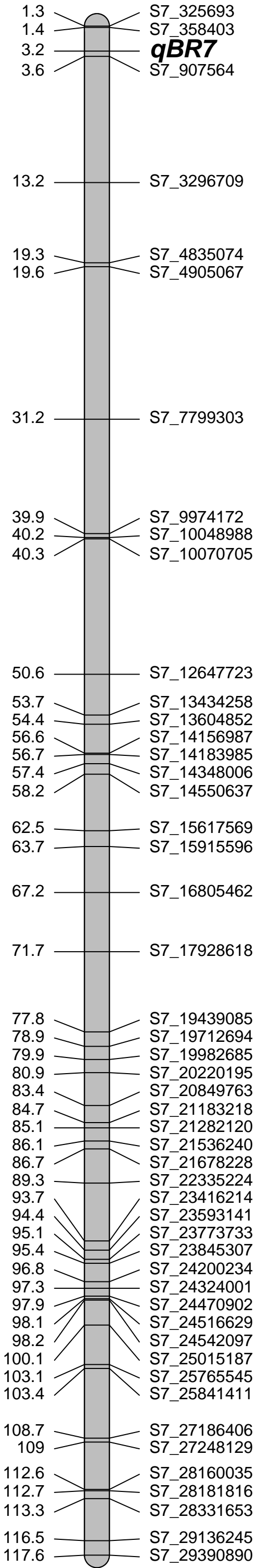

Chr. 8

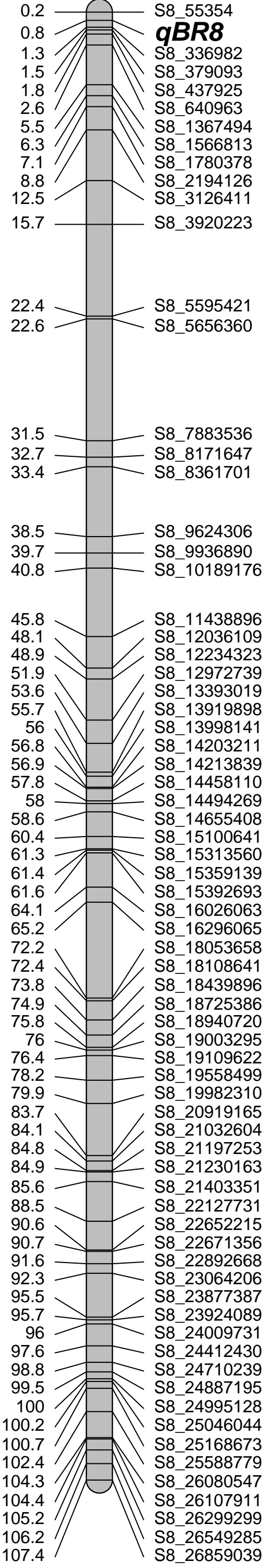

Chr. 11

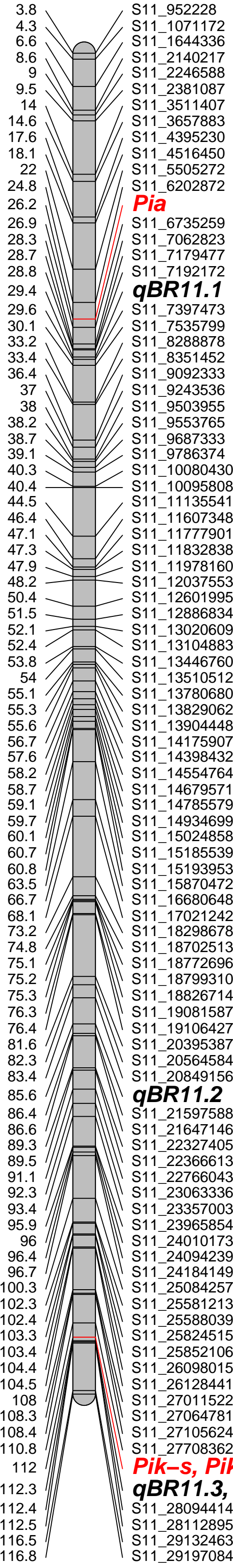

Chr. 12

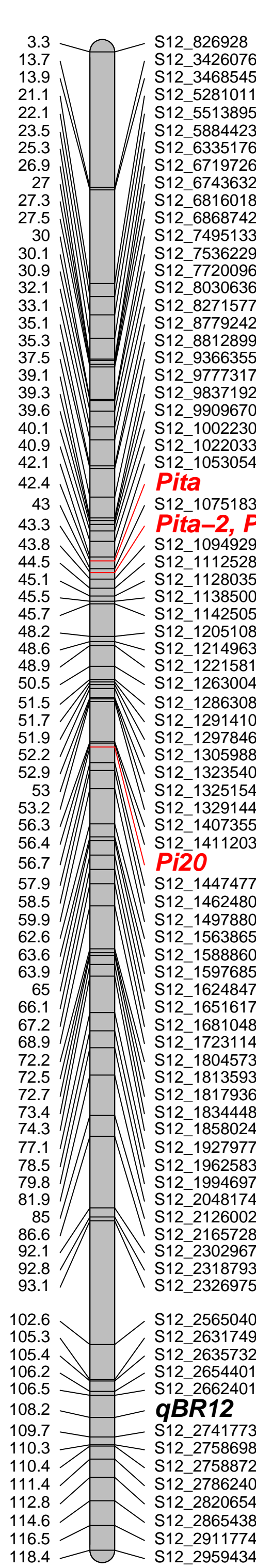

Supplement: Supplementary file 1 [file plants-12-00863-s001.zip › Figure S1.pdf]
